# The Effect of Melatonin on Mitochondrial Function and Autophagy in *In Vitro* Matured Oocytes of Aged Mice

**DOI:** 10.22074/cellj.2020.6302

**Published:** 2019-09-08

**Authors:** Zahraa Nasheed Hamad Almohammed, Fatemeh Moghani-Ghoroghi, Iraj Ragerdi-Kashani, Rouhollah Fathi, Leila Sadat Tahaei, Mohamad Naji, Parichehr Pasbakhsh

**Affiliations:** 1 Department of Anatomy, School of Medicine, Tehran University of Medical Sciences, International Campus, Tehran, Iran; 2 Department of Gynecology, Alshatra Hospital, Thiqar Health Office, Health Ministry of Iraq; 3 Department of Embryology, Reproductive Biomedicine Research Center, Royan Institute for Reproductive Biomedicine, ACECR, Tehran, Iran; 4 Department of Embryology, Reproductive Biomedicine Research Center, Royan Institute for Reproductive Biomedicine, ACECR, Tehran, Iran; 5 Urology and Nephrology Research Center, Shahid Beheshti University of Medical Sciences, Tehran, Iran

**Keywords:** Aged Mice, Autophagy, Melatonin, Mitochondria

## Abstract

**Objective:**

This study examined the *in vitro* effect of melatonin on the protein synthesis of mitochondria, as well as autophagy in matured oocytes of aged mice.

**Materials and Methods:**

In this experimental study, germinal vesicles (GV) oocytes were collected from aged (with the age of six-months-old) and young mice (with age range of 6-8 weeks old) and then cultured in the *in vitro* culture medium (IVM) for 24 hours to each metaphase II (MII) oocytes and then supplemented with melatonin at a concentration of 10 μM. The culture medium of MII oocytes was devoid of melatonin. Afterward, the expression of the SIRT-1 and LC3 was assessed by immunocytochemistry. ATP-dependent luciferin-luciferase bioluminescence assay was employed for the measurement of the ATP contents. Intracellular reactive oxygen specious (ROS) was detected by DCFH-DA, and the total antioxidant capacity (TAC) level was determined by TAC assay.

**Results:**

The expression of SIRT-1 and LC3, as well as the measurement of the ATP content, was significantly increased in oocytes treated with melatonin compared with the oocytes receiving no treatment. Moreover, TAC was considerably higher in melatonin-treated oocytes than oocytes receiving no treatment. On the other hand, the level of ROS was significantly decreased in oocytes treated with melatonin in comparison with the untreated oocytes. The results indicated that melatonin considerably improved the development of oocytes as well.

**Conclusion:**

According to the data, melatonin increased mitochondrial function and autophagy via an increase in the expression of SIRT1 and LC3, as well as the ATP contents while it decreased the levels of ROS and increased TAC in oocytes derived from aged mice.

## Introduction

Age-related infertility is one of the significant concerns of female individuals (1). In 1975, only 5% of pregnant women were over 30 years old, whereas this percentage was increased up to 26% in 2010 (2). Although aging influences all features of female reproduction, most studies have focused on oocytes (3). Several lines of evidence demonstrated that aging alters both the quality and quantity of oocytes (4). The precise mechanism underlying age-induced reproductive disorders is still unclear; however, hormonal imbalance, reduced ovarian follicle reserve, increased oocyte aneuploidy, and mitochondrial dysfunction in oocytes are involved in this scenario (5). The main factor restricting the success rate of assisted reproduction techniques (ART) is oocyte competence. Although ART has been widely improved, the percentage of successful pregnancies and alive babies are 47.7 % for women younger than 35 and less than 30% for women older than 35 (6, 7).

Several studies have reported a relationship between oocyte quality and mitochondrial function (8). The number of mitochondria and their function are regulated through the organized processes of mitochondrial biosynthesis and degradation in the cells (9). SIRT is a vital mitochondrial deacetylase, which regulates biological mitochondrial functions (10). 

SIRT-1 is associated with the regulation of autophagy and mitochondrial function in the cells which can increase the ATP contents within the cells and protect them from excessive reactive oxygen species (ROS) and oxidative damage (11). 

Autophagy is a cellular process that leads to the degradation and removal of damaged organelles mediated by lysosomes. It has been implicated that melatonin improves mitochondrial functions (12). 

LC3 is a protein marker, located on the membrane of the autophagosome (9). Mitochondrial functions in oocytes can be affected by excessive ROS. So, the ROS concentration should be counterbalanced by the activity of antioxidant agents (13).

Melatonin (N-acetyl-5 methoxytryptamine) has been introduced as a free radical scavenger and could be indirectly considered an antioxidant molecule (14). Hence, the use of melatonin for the decrease of age- related mitochondrial oxidative stress in oocytes could be a point of view. Since aging is associated with low oocyte competence and infertility, the current study was designed to evaluate whether melatonin can improve the quality of aged oocytes thereby increasing the mitochondrial number and protein synthesis, as well as the ATP contents of aged murine oocytes during *in vitro* culture medium (IVM). Our results provide influential perceptions into the mechanisms of aging and mitochondrial regulation in oocytes. 

## Materials and Methods

All chemicals in this experimental study were purchased from Sigma (St Louis, MO, USA) except for fetal calf serum (FCS) which was obtained from Invitrogen (Carlsbad, CA, USA). Human chorionic gonadotropin (hCG) and follitropin alfa (Gonal-F) were procured from Organon (Oss, Netherlands). 

### Animal procedures

NMRI mice (purchased from the Pasteur Institute of Iran) were housed in an air-conditioned room under a 12 hours light: 12 hours dark cycle (7 AM to 7 PM) and temperature 20-25°C with free access to food and water. All animal experiments were carried out according to the guidelines of the Iranian Council for Use and Care of Animals and approved by the Animal Research Ethical Committee of Tehran University of Medical Sciences (Ethical Committee code: IR. TUMS.VCR. REC.1397.4954). 

### Experimental groups

All experiments were carried out in two main groups as follows; the first group consisted of young mice with age range of 6-8 weeks (15-17) and the second group included old mice with the age of six months (18). Female NMRI mice received an intraperitoneal injection of 5 IU pregnant mare serum gonadotropin (PMSG). Then mice were sacrificed by cervical dislocation 48 hours after the injection of PMSG and ovaries were collected and transferred to a petri dish containing the α-MEM culture medium supplemented with 5% fetal bovine serum (FBS) and a mixture of antibiotics (penicillin, streptomycin). Oocytes at the germinal vesicle (GV) stage were mechanically isolated from ovaries and collected under a stereomicroscope (Nikon SMZ- 2T, Japan). 

### *In vitro* maturation of germinal vesicle oocyte

The *in vitro* maturation medium consisted of the a-Minimum Essential Medium (α-MEM, Sigma, USA) supplemented with 5 mg/ml streptomycin, 6 mg/ ml penicillin, 5% fetal calf serum (FCS, Invitrogen, USA), 100 mIU/ml recombinant human follicle stimulating hormone (rhFSH), and 7.5 IU/ml human chorionic gonadotropin (hCG, Sigma, USA) . The GV stage oocytes (n=6-8) were cultured with 0 or 10 µM melatonin (19) at 37°C, 5% CO_2_ and 95% humidity in a 20-µl drop of the IVM medium for 24 hours in both old and young groups. After 24 hours of the culture period, the maturity of the oocytes in the above groups was assessed under an inverted microscope (Labamed, USA). Oocytes which reached to the MII stage were selected for further experiments. 

### Detection of SIRT1 and LC3 by fluorescence immunostaining 

After 24 hours of the culture period in the IVM medium with 0 or 10 µM melatonin, five MII stage oocytes were randomly chosen from each young and old groups and then the immunofluorescence experiments were performed (20). After removal of zona pellucida by Tyrod’s acid solution (Sigma-Aldrich, USA), oocytes were fixed and permeabilized with 4% paraformaldehyde with 0.1% Triton X-100 in phosphate-buffered saline (PBS, Sigma, USA) for 20 minutes at room temperature, then washed with 0.3% Triton X-100 in PBS for 5 minutes. Afterward, oocytes were blocked in a 10% bovine serum albumin (BSA, Sigma, USA)/PBS drop for 30 minutes. Finally, they were incubated with a primary antibody containing anti-LC3 and anti-Sirt1 [rabbit polyclonal, 1:100 (Abcam, USA)] in 2% BSA/PBS at 4°C overnight. In the next day, oocytes were washed three times in 2% BSA/PBS and incubated with fluorescein-conjugated goat anti-rabbit IgG (1:200; Abcam, USA) as a secondary antibody for at 37°C for 40 minutes. After three times washing by PBS, oocytes were mounted on glass slides using an anti- fade reagent containing 6-Diamidino-2-phenylindole (DAPI, Sigma-Aldrich, USA). The expression of SIRT1 and LC3 was evaluated using a fluorescence microscope (Labamed, USA) at 488-excitation wavelengths. The images of individual oocytes in each group were captured by a digital camera (DeltaPix, Denmark). The fluorescence intensity of each marker was quantified using the Image J (1.48. version) software (National Institutes of Health, Bethesda). 

### ATP quantification

The measurement of the ATP content of oocytes was carried out using the luminescence (Berthold LB 9501 illuminometer) generated in an ATP-dependent luciferinluciferase bioluminescence assay. A commercial ATP assay kit (ATP bioluminescence assay kit HS II Roche) was used following the procedure defined by the manufacturer’s recommendations. A total of 35-50 MII stage oocytes from each group was mixed with 50 ml of lysis solution and vortexed for one minute on ice for the lysis process. Then, the mixture was centrifuged at 12,000 g at 4°C for 10 minutes, and the supernatant was applied for further assessments. A six-point standard curve (0-5 pmol) was deliberated in each series of an assay. The standard curves were generated, and the ATP content was calculated using the formula derived from the linear regression of the standard curve. 

### Determination of Intracellular reactive oxygen species

To quantify of ROS levels, 40-50 MII stage oocytes from each group were incubated with 2 µM of 2’,7’-dichlorofluorescein diacetate (DCFH-DA, Sigma, USA) at 37°C for 30 minutes in the αMEM medium in a dark place (21). After 3 times washing with αMEM, oocytes were analyzed under a fluorescence microscope (Olympus BX51, Japan) equipped with UV filters (450490 nm (excitation) and 520 nm (emission) filters. The fluorescence intensity of oocytes was assessed by the ImageJ (1.48. version) software (National Institutes of Health, Bethesda). 

### Measurement of total antioxidant capacity content

Oocytes at the GV stage were cultured in the IVM culture medium for 24 hours. After 24 hours, 50 µL of the culture medium from each group was collected for the measurement of the TAC content. A commercial kit (Zell Bio GmbH, Germany) was used for the quantitative assay of TAC by the oxidation-reduction colorimetric assay. All of the procedures were performed according to the manufacturer’s instruction. Then, the TAC concentration (mM) in samples was calculated based on the standard curve drawn using the standard optic density absorbance against the standard concentration. TAC concentration was determined in the range of 0.125-2 mM. 

### Statistical analysis

All experiments were performed in triplicate, and the data were expressed as the mean ± standard deviation (SD). The statistical analysis was carried out using one- way analysis of variance (ANOVA) followed by Tukey’s post hoc tests using the SPSS 16 version. The P<0.05 was considered statistically significant. 

## Results

### Effect of Melatonin on SIRT-1 expression

**Fig.1:**
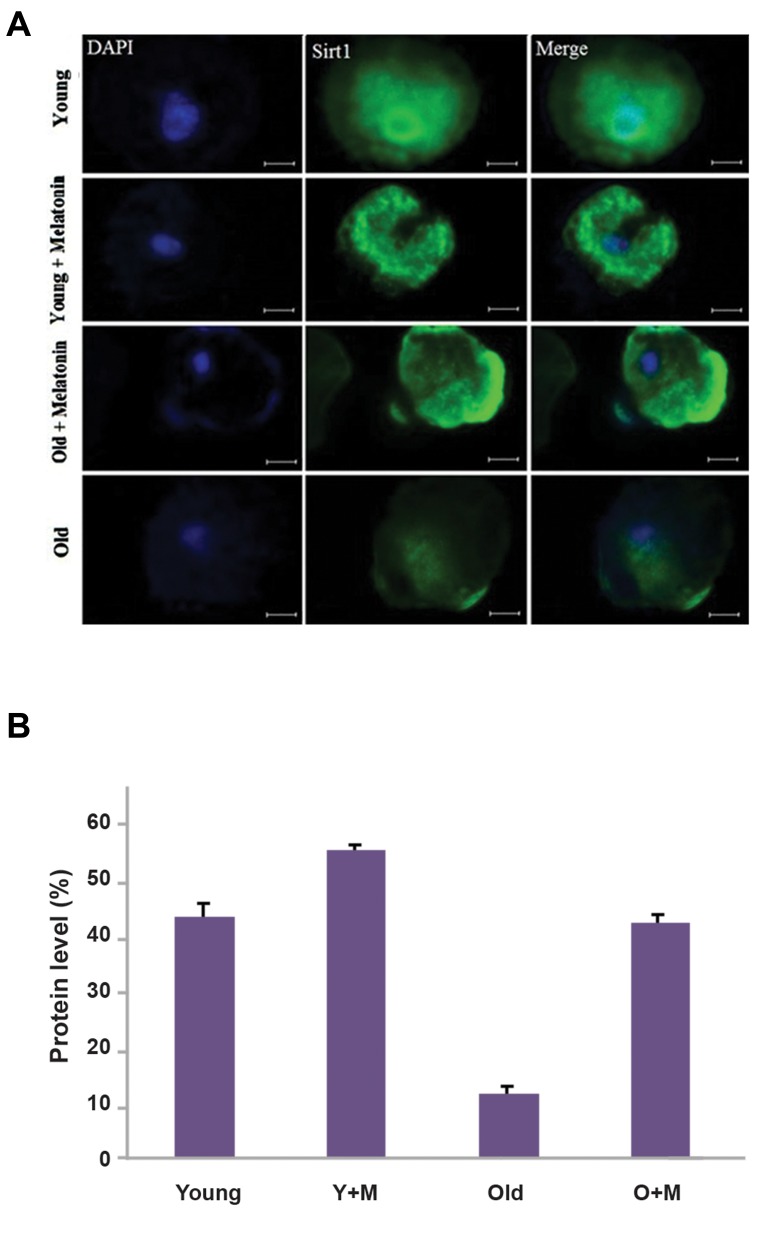
The expression of SIRT-1 at the MII stage of *in vitro* matured oocytes, isolated from young and aged mice was evaluated usingimmunofluorescence staining. **A.** The micrograph represents the intensity of the SIRT-1 expression among the young MII oocyte, young MII oocyte+melatonin, aged MII oocyte+melatonin, and aged MII oocyte groups. The nuclei were stained by DAPI. The secondary antibody was conjugated with FITC and **B.** The expression of SIRT-1 in the aged MII oocyte+melatonin group was significantly higher than the aged MII oocyte (P<0.01). Accordingly, the SIRT-1 expression was elevated in the young MII oocyte+melatonin group compared with the young MII oocyte group (P<0.05) (magnification × 400, scale bars: 20 µm). Y+M; Young MII oocyte+melatonin and O+M; Aged MII oocyte+melatonin.

The immunostaining analysis was performed to evaluate the effects of melatonin on the expression of SIRT-1 in oocytes. The results of immunostaining following the treatment with melatonin showed that 10 µM melatonin upregulated the SIRT-1 expression in the aged MII oocyte+melatonin group versus the aged MII oocyte group (42.2 ± 0.99% vs. 11.9 ± 0.54% respectively, P<0.01). Moreover, a higher expression of SIRT-1 was observed in the young MII oocyte+melatonin group compared with the young MII oocyte group (54.4 ± 1.65% vs. 42.8 ± 3.34, respectively, P<0.05). As shown in Figure 1, there was no significant difference between the aged MII oocyte+melatonin group and young MII oocyte group (42.2 ± 0.99% vs. 42.8 ± 3.34%, respectively, P=0.84). 

### Effect of melatonin on autophagy in oocytes

We examined the expression of the LC3 protein (the marker of autophagosomes) in oocytes by the immunostaining method to assess the effect of melatonin on autophagy. 

**Fig.2:**
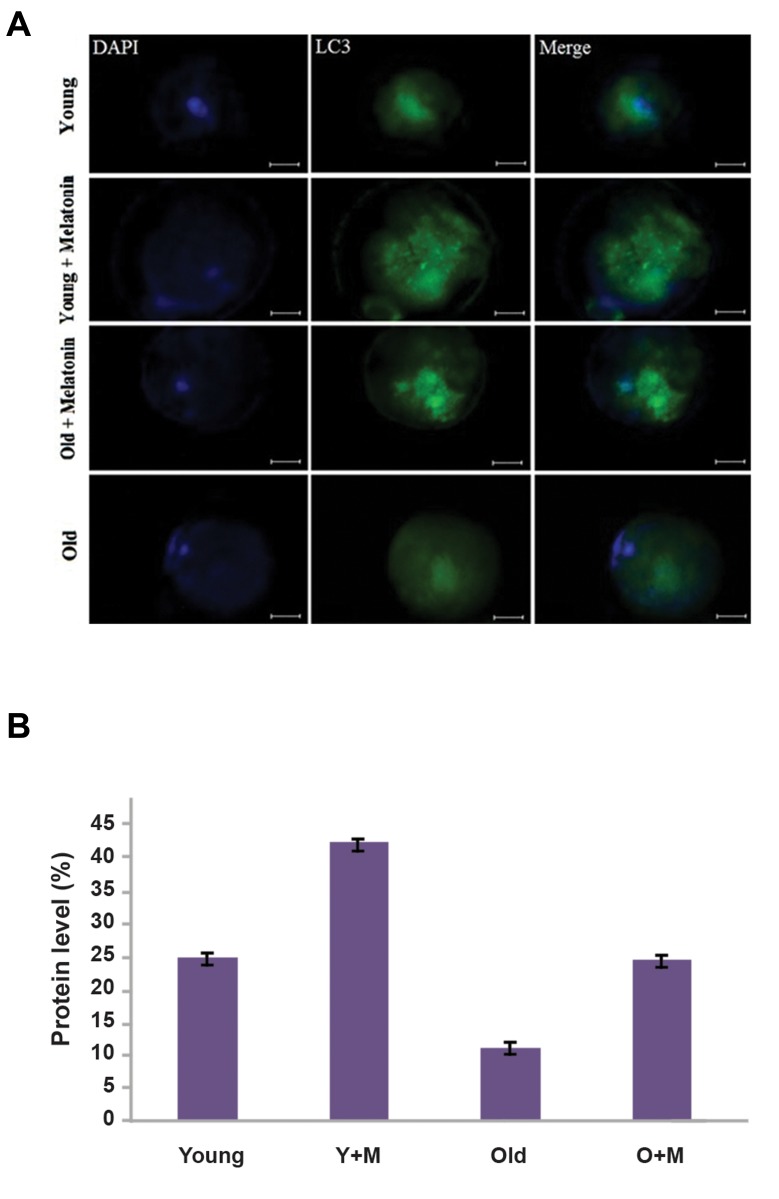
The expression of the LC3 protein in *in vitro* matured MII oocytes, isolated from aged and young mice was determined by the Immunofluorescence staining. **A.** The micrograph represents a significant difference in intensity of the LC3 expression between the young MII oocyte, young MII oocyte+melatonin, aged MII oocyte+melatonin, and aged MII oocyte groups. The nuclei were stained by DAPI. The secondary antibody was conjugated with FITC (magnification ×400, scale bars: 20 µm) and **B.** Significantly higher levels of LC3 were found in the aged MII oocyte+melatonin compared with the aged MII oocyte groups (P<0.01). The expression of the LC3 was significantly higher in the young MII oocyte+melatonin than the young MII oocyte groups (P<0.01). Y+M; Young MII oocyte+melatonin and O+M; Aged MII oocyte+melatonin.

The expression of the LC3 protein in oocytes has been shown in Figure 2. The results indicated that LC3 was significantly upregulated in the aged oocyte+melatonin group versus the aged oocyte group (24.1 ± 0.37% vs. 11.05 ± 1.25%, respectively, P<0.01). Also, significantly higher expression of the LC3 protein was observed in young oocyte+melatonin group versus the young MII oocyte group (42.06 ± 0.26% vs. 24.81 ± 0.7%, respectively, P<0.01). As depicted in Figure 2, our data showed that there was no significant difference between the aged MII oocyte+melatonin group and young MII oocyte group (24.1 ± 0.37% vs. 24.81 ± 0.7%, respectively, P=0.36). 

### Effect of Melatonin on the ATP content of *in vitro* matured oocytes

**Fig.3:**
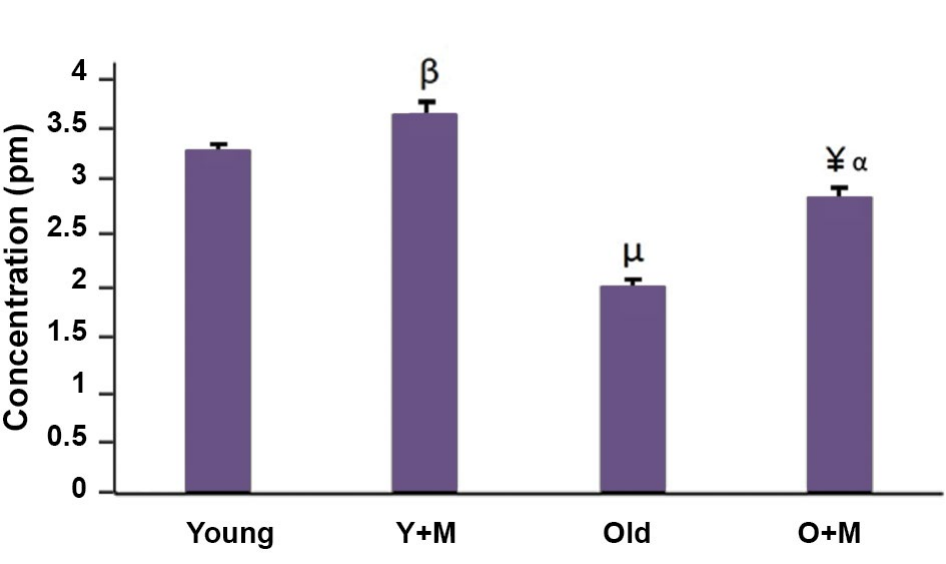
The levels of the ATP contents of *in vitro* matured MII oocytes in all experimental groups, namely aged MII oocyte, young MII oocyte, aged MII oocyte+10 µM melatonin, and young MII oocyte+10 µM melatonin. Each group consisted of 35-50 MII oocytes. The obtained data were represented as mean ± SD. ¥; P<0.001 vs. aged group, ß; P<0.05 vs. young group, a; P<0.01 vs. young group, µ; P. 0.001 vs. young group, Y+M; Young+melatonin, and O+M; Aged+melatonin.

The effect of melatonin on the ATP content of in- vitro matured oocytes was assessed by ATP-dependent luciferin-luciferase bioluminescence assay. The levels of ATP were compared among different groups in Figure 3. The data showed that the ATP levels were significantly increased in the aged MII oocyte+melatonin group in comparison with the aged MII oocyte group (2.7 ± 0.1 vs. 1.9 ± 0.07 pmol, respectively, P<0.001).

Moreover, the ATP contents of the young MII oocyte+melatonin group were significantly higher than the young MII oocyte group (3.5 ± 0.1 vs. 3.1 ± 0.1 pmol, P<0.05). As indicated in Figure 3, there was a significant difference between the aged MII oocyte+melatonin and young MII oocyte group as well (2.7 ± 0.1 vs. 3.1 ± 0.1 pmol, respectively, P<0.01). 

### Melatonin increased total antioxidant capacity in culture media of *in vitro* matured oocytes 

**Fig.4:**
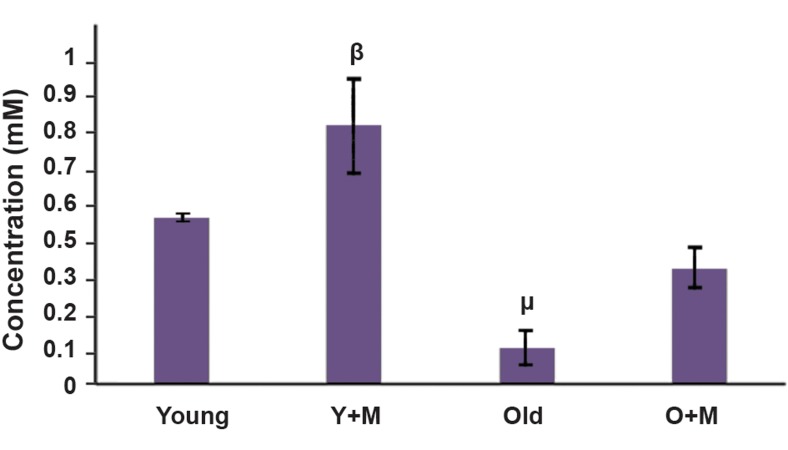
The total antioxidant capacity (TAC) of MII stage *in vitro* matured mouse oocytes in four groups: old and young or old and young supplemented by 10 µM melatonin. 50 µL of culture media of each group were used for TAC content measurement. The data was represented based on mean ± SD. Although TAC level increased in aged MII oocyte+melatonin in comparison to the aged MII oocyte group, (0.35 ± 0.06 vs. 0.11 ± 0.05 mM) but there is no significant difference between them (P=0.07). The result shows that there is no significant difference between aged MII oocyte+melatonin and Young groups as well (P=0.31). It also shows a significant difference between young MII oocyte+melatonin vs. young MII oocyte group. µ; P<0.01, ß; P<0.05, Y+M; Young MII oocyte+melatonin, and O+M; Aged MII oocyte+melatonin.

TAC was measured in culture media of *in vitro* matured oocytes to monitor the efficacy of melatonin in antioxidant capacity of oocytes. The results of TAC levels in different groups are shown in Figure 4. As demonstrated in Figure 4, the level of TAC was increased in the aged MII oocyte+melatonin group in comparison with the aged MII oocyte group (0.35 ± 0.06 vs. 0.11 ± 0.05 mM), but there was no significant difference between them (P=0.07). The TAC level was also significantly higher in the young MII oocyte+melatonin group compared with the young group (0.79 ± 0.14 vs. 0.51 ± 0.00, respectively, P<0.05). Moreover, the results demonstrated that there was no significant difference between the aged MII oocyte+melatonin and young MII oocyte group (P=0.31). 

### Melatonin decreased the reactive oxygen species level in *in vitro* matured MII oocytes 

**Fig.5:**
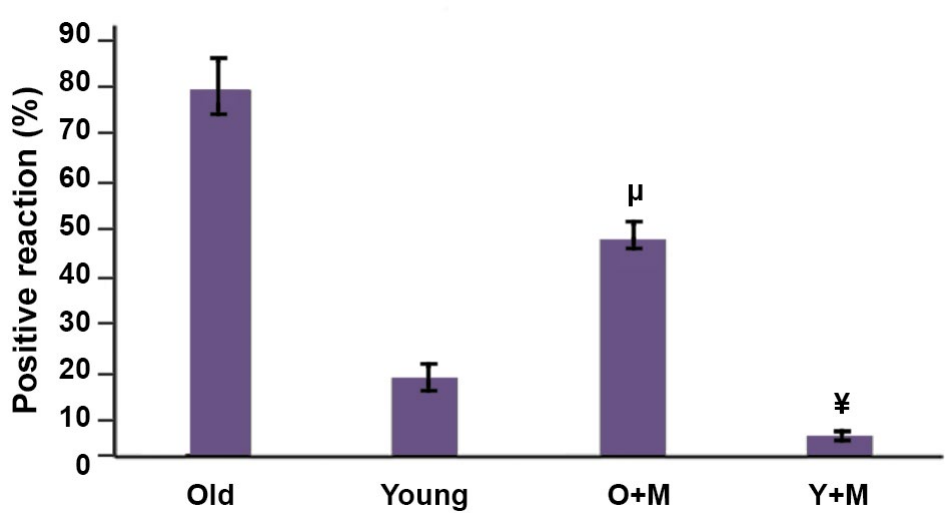
Intracellular reactive oxygen specious (ROS) levels of MII *in vitro* matured oocytes were measured by immunofluorescence dye (DCFHDA) in all experimental groups, namely aged MII oocyte, young MIIoocyte, aged MII oocyte+10 µM melatonin, and young MII oocyte+10µM melatonin and they were quantified by the ImageJ software. Eachgroup consisted of 40-50 MII oocytes. The results were expressed asmean ± SD. The different symbols represent a significant differencebetween the two experimental groups. Although the ROS level wasdecreased in young MII oocyte+melatonin group compared with theyoung MII oocyte group, the difference was not statistically significant(4 ± 0.81 vs. 17 ± 3.09, P=0.71). The results also showed that there wasno significant difference between the aged MII oocytes+melatoninand young MII oocytes groups (P=0.10). ¥; P. 0.05 vs. aged group, µ; P<0.001 vs. young group, Y+M; Young+melatonin, and O+M; Aged+melatonin.

**Fig.6:**
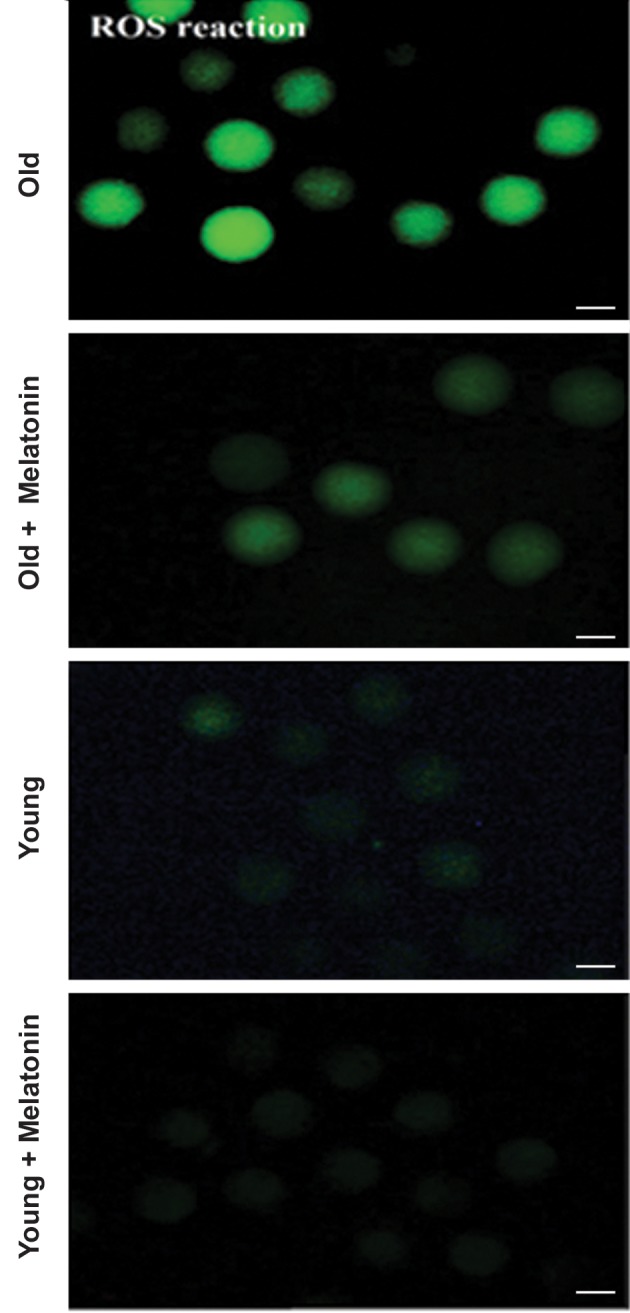
The levels of DCFH-DA representing the reactive oxygen specious (ROS) production in MII *in vitro* matured oocytes, isolated from young and aged mice. The micrograph depicts the different intensity of ROS among the young MII oocytes, young MII oocytes+10 µM melatonin, aged MII oocytes, and aged MII oocytes+10 µM melatonin groups. The phase contrast of each group shows the morphology of oocytes. The fluorescence intensity of DCFH-DA was applied to probe ROS within the cytoplasm of oocytes (magnification: ×200, scale bars: 100 µm).

The rate of oxidative stress in oocytes was evaluated by the measurement of intracellular ROS using DCFH-DA. The levels of ROS in different groups are illustrated in Figures 5 and 6. The increased production of ROS was markedly reversed upon the treatment with melatonin. The results show that the fluorescence intensity of stained oocytes with DCFH-DA in the aged MII oocyte+melatonin group was significantly lower than the aged MII oocyte group (47 ± 3.09 vs. 79 ± 6.18, respectively, P<0.05). Although the ROS level was decreased in the young MII oocyte+melatonin compared with the young MII oocyte group, the difference was not statistically significant (4 ± 0.81 vs. 17 ± 3.09, respectively, P=0.71). Moreover, there was no significant difference between the aged MII oocyte+melatonin and young MII oocyte groups (P=0.10). 

### Melatonin improved the development of *in vitro* matured oocytes

A total of 680 oocytes at the GV stage were used for *in vitro* maturation. Meiotic competency of oocytes among the different groups was determined after 24 hours of the *in vitro* maturation process. Percentage of MII oocytes in the aged MII oocyte+melatonin group was 80.12%, which was significantly higher than the aged MII oocyte group (63.63%, P<0.001). There was a significant difference between the young MII oocyte+melatonin and young MII oocyte groups (92.34 and 70.17% respectively, P<0.0001). The results also showed that there was a significant difference between the aged MII oocyte+melatonin and young MII oocyte groups (P<0.05). 

## Discussion

Reproductive senescence has been introduced as a major health problem over the world. Female fertility is promptly decreased after age of 35 years. A decline in ovarian follicle reserve and oocyte pool, as well as an increase in the number of low-quality oocytes, are featured characteristics of ovarian aging (22). Perhaps, diminished mitochondrial biogenesis has been regarded as a significant factor related to poor oocyte quality as a result of aging (23). Although the mechanisms underlying age-induced decreased oocyte quality is still unknown, mitochondrial dysfunction is thought to be involved in this process (3). Various antioxidants such as resveratrol were found to improve mitochondrial function through the activation of SIRT-1 (24, 25). 

Melatonin is an effective antioxidant and free-radical scavenger which has a central role in the improvement of ovarian function and oocyte quality (26). It has been reported that melatonin supplementation significantly postpones postovulatory aging of murine oocytes through the upregulation of the expression of SIRT-1. It has been reported that melatonin could reverse age-induced reproduction damage caused by postovulatory aging through the regulation of the SIRT-1 expression (27). Our results also showed that the culture of oocytes, which were at the GV stage, with melatonin for 24 hours considerably enhanced the expression level of SIRT-1 in oocytes in both aged and young mice. Melatonin could increase the SIRT1 expression in aged MII oocyte+melatonin as much as the young MII oocyte group, implying the improvement of mitochondrial function.

It has been reported that SIRT-1 is also associated with the regulation of autophagy, a cellular process that ends with lysosomal degradation, and mitochondrial activities in cells upon oxidative stress (28, 29). Autophagy is a process that degrades misfolded and long-lived proteins and damaged organelles such as mitochondria, endoplasmic reticulum, as well as intracellular pathogens, to maintain cellular homeostasis (12, 29). 

The LC3 protein which is generally localized on autophagosome membranes can be considered a biomarker of autophagy. In a previous study, it has been reported that resveratrol significantly increased autophagosomes in oocytes of aged cows and enhanced oocyte competence (9). On the other hand, the results of another research showed that melatonin attenuated autophagy in postovulatory oocytes (27).

Our results demonstrated that melatonin could significantly increase the LC3 expression in oocytes of aged mice, indicating an increase in the number of autophagosomes. Moreover, the LC3 expression in aged MII oocyte+melatonin group had no significant difference when compared with the young MII oocyte group, showing that melatonin could increase the number of autophagosomes similar to that of the young MII oocyte group.

Mitochondrial involvement in the aging process is also attributed to the energy production and regulation of the different cellular signaling pathways (30). Adenosine triphosphate is mainly produced in mitochondria, and it is essential for oocytes. The ATP generation is one of the major tasks of mitochondria, and the amount of ATP in mature oocytes represents the quality of oocytes (31). The level of ATP in oocytes could be considered an indicator of the developmental potential of mammalian oocytes (26). According to the literature, poor oocyte quality and failure in embryonic development could be directly associated with the sub-normal production of ATP (32). 

Although increased ROS production in aged oocytes has been shown to result in a decrease in the concentrations of intracellular ATP (26), other scientists believe that SIRT could increase the ATP level and thus protecting the cells from ROS-mediated oxidative damage (11). Melatonin can improve mitochondrial function by an increase in the ATP production within oocytes (33).

In the present study, it has been found that *in vitro* matured melatonin-treated oocytes of old and young mice exhibited a significant increase in ATP content compared with those untreated oocytes. It is suggesting that melatonin could enhance mitochondrial function.

Notably, the comparison between melatonintreated and untreated oocytes revealed that there was a significant increase (1.4 fold) in the ATP content in the aged MII oocyte+melatonin group as compared with the young MII oocyte group (increased by 1.1 fold). A significant difference observed between the aged MII oocyte+melatonin and young MII oocyte groups indicated that although melatonin increased the ATP content in the aged MII oocyte+melatonin group, such an increase did not reach to that of the young MII oocyte group.

Considering the primary source of ROS production is placed in mitochondria, the aging process increases the rate of mitochondrial ROS (mROS) and weakens antioxidant defense systems (22, 34). Scientists believe that mitochondria have a critical role in cellular events associated with the aging process, through an accumulation of mitochondrial ROS and oxidative damage to mitochondrial and cytoplasmic components. According to various theories, mitochondrial respiratory activity and mitochondrial membrane potential are diminished during the aging process and endogenous antioxidant system function, denoting that these phenomena are decreased in an age-dependent manner (22). Therefore, to reduce the adverse effects of excessive ROS and improve the maturation process of oocytes, antioxidants are widely used in *in vitro* culture systems (35). Melatonin is an effective modulator of mitochondrial DNA damage. It has been implicated that an increase in the electron transport efficiency within mitochondria prevents ROS formation and protects DNA mutation in response to oxidative damage (36).

Some reports have indicated that melatonin and its metabolic derivatives can consecutively detoxify ROS and regulate different antioxidant enzymes through their receptors to halt radical-mediated damages, leading to preservation of the quality of oocytes (37). Melatonin could also dramatically decrease the ROS level in porcine oocytes and improve the quality of oocytes (38).

In this study, we found that the addition of melatonin to the culture medium significantly reduced the ROS level in oocytes and increased TAC in the culture media. Based on above statements, it would be plausible that melatonin not only reduces ROS level via its direct ROS-scavenging ability but also improves the mitochondrial function by the enhancement of autophagy which maintains cellular homeostasis and oocyte quality.

Previous studies demonstrated that melatonin supplementation during the in-vitro culture significantly reduced ROS production and augmented the glutathione (GSH) contents (39). Our findings were inconsistent with other studies which report that melatonin has a direct protective effect against oxidative stress for mammalian oocytes. Although our data showed that melatonin increased TAC levels in the aged MII oocyte+melatonin group in comparison with the aged MII oocyte group, there was no significant difference between the two groups. On the other hand, the TAC level was increased significantly in the young MII oocyte+melatonin group compared with young oocyte group. It has also been observed that there was no significant difference between the aged oocyte+melatonin and young oocyte groups, implying that melatonin improved the ability of aged MII oocytes to increase the level of TAC in comparison with the ability of the young MII oocytes.

Melatonin also improved the oocyte maturation rate and consequently the embryo development thereby the reduction of ROS during the *in vitro* maturation process of the porcine oocyte (39). Our results showed a significant increase in meiotic competency of melatonin-treated MII oocytes in comparison with non-treated oocytes. 

## Conclusion

The present study demonstrated that the treatment of *in vitro* matured MII oocytes, isolated from aged mice with melatonin could improve the mitochondrial function by an increase in the SIRT-1 expression, the ATP content, and autophagy, ultimately resulting in the improvement of the quality of mitochondria in cells. Melatonin also decreased intracellular ROS and increased TAC production. As the final point, melatonin could improve the oocyte maturation rate.
